# Response to Weiss HR, Moramarco M: “Indication for surgical treatment in patients with adolescent idiopathic scoliosis – a critical appraisal” (*Patient Saf*. *Surg*. 2013, 7:17)

**DOI:** 10.1186/1754-9493-7-26

**Published:** 2013-07-18

**Authors:** Shay Bess

**Affiliations:** 1 Rocky Mountain Scoliosis and Spine, Rocky Mountain Hospital for Children and Presbyterian/St Luke’s Medical Center, 2055 High Street, Suite 130, 80205 Denver, CO, USA

## 

In a letter to the editor published recently in the *Journal* (Weiss HR and Moramarco M, *Patient Saf*. *Surg*. 2013, 7:17), the authors provide an anecdotal selection and interpretation of the current literature on complication rates, revision surgery rates and outcomes of surgical treatment for adolescent idiopathic scoliosis (AIS). They conclude that AIS is a “relatively benign” disease state and the “long-term outcome of surgery for AIS creates a more negative end result over the course of a lifetime than the natural history of the condition itself.” The authors’ interpretation of the literature is unfortunate and misguided, and, as a consequence, provides a biased, single-sided view of the AIS disease state and attempts to refute surgery as a viable treatment option for patients affected with AIS. With any disease state, treating physicians should strive to remain open minded and educated as to the current literature regarding the diagnosis, treatment options and treatment outcomes, and should integrate this information into their practice and share this information with the patients and families that they treat in a factual and unbiased manner. This in turn will facilitate educated discussions between the patient and the physician so that the treatment that is mutually agreed upon is consistent with the existing science and reflects the patient’s desires. The article does not provide a current literature review of the literature. The majority of the basic science, natural history and treatment outcomes literature sited in this “review article” does not reference the original manuscripts, but instead refers to review articles previously written by the senior author (HRW). These review articles carry the same tone and biased data interpretation as the current article, therefore the reader is unable to directly reference the true source of the information, prohibiting an independent interpretation of the information provided. In light of the aims of this review, however it is worth truly investigating what the current literature reports regarding the health impact of AIS and the outcomes following treatment. Loner, et al. compared the health-related quality of life (HRQOL) measures for patients with Scheuermann's kyphosis (SK), AIS, and normal adolescent populations using the SRS-22 outcomes instrument and Visual Analogue Pain Scale (VAS). [1] patients affected with spinal deformity reported worse baseline HRQOL scores for all domains except mental health. AIS patients reported greater difficulty with activities, worse self-image and worse total SRS-22 scores than normal controls. Pellegrino et al. performed a prospective observational study to assess patient quality of life before and after surgical treatment of AIS and evaluate the association between quality of life and curve magnitude, curve correction, and type of instrumentation used in surgery. At one year follow up, SRS-30 and SF-36 scores improved significantly. The greatest changes occurred in the Self-Image and Satisfaction with Management domains of the SRS-30 survey. Total SRS-30 scores were significantly improved at 6- and 12-month follow-up, as were sub-scores in the general health, vitality, and social functioning domains of SF-36 [2]. These results are consistent with previous data indicating AIS has a measurable, negative impact on HRQOL when patients with AIS are compared to controls, and that surgical treatment of AIS can improve reported HRQOL [3-14].

Regarding the original research that the authors do cite, Mueller et al. reported on 40 surgically treated AIS, the long-term follow up rate was 35%. The authors reported a 25% rate of late onset infection and 20% incidence of implant removal due to implant related pain [15]. These results are exceedingly high and are not consistent with reported values. A recent literature review points to rates of acute infections for AIS ranging from 0.5-6.7% and late onset infection (>12 months) ranging from 2.77- 4.7% [16-21]. Risk factors for late infection include use of stainless steel implants, and prominent implants. The most common pathogens are *S*. *epidermidis* and *Propionibacterium acnes*. Surgeon and/or institution contamination by these pathogens is a known risk factor. All of these values point to the report by Mueller as an outlier. The reference by Westrick, et al. refers to a systematic review evaluated the scientific evidence on the long-term outcomes and complications of surgical intervention for AIS. The authors concluded that surgery reliably arrests the progression of deformity, achieves permanent correction, and improves appearance however, no long-term, prospective controlled studies exist to support the hypothesis that surgical intervention for AIS is superior to natural history. [22]. This study highlights several limitations within the literature, including poorly performed historical studies with incomplete follow up and poor metrics used to collect HRQOL outcomes, as well as the lack of prospective, randomized studies for AIS, which for humanitarian reasons have been and always will be very difficult to perform. Reading the article by Westrick et al. in this light, sheds insight to the challenges of high quality research. The data interpretation of this article by HRW and MM also typifies the misinterpretation that the authors HRW and MM because their interpretation of the current literature is jaded by a zealous rejection of surgery for AIS.

In conclusion, the authors unfortunately miss the potential value of systematic review for treatment options for AIS. Instead the authors use the current literature as a means to promote their own agenda. Observation, physical therapy, bracing and surgery all have a distinct role for effectively treating the AIS patient and each modality should be appropriately indicated based upon the patient’s deformity magnitude, general health, level of function and satisfaction, as well as the patient’s and the parents’ treatment desires. To neglect the established value of any of these treatment options stands as a detriment to patient care.

## Competing interests

Consultant: Depuy Spine, Medtronic, K2.

Research support: Depuy Spine, Medtronic.

Scientific Advisory Board: Allosource.

Royalties: Pioneer Spine.
